# The relationship between personality traits, sex, age, performance level, and playing position in recreational handball

**DOI:** 10.1186/s40359-026-04778-x

**Published:** 2026-05-23

**Authors:** Marc Niering, Janine Körling, Rainer Beurskens, Johanna Seifert

**Affiliations:** 1 Department of Psychiatry, Social Psychiatry, and Psychotherapy, Hannover Medical School, Carl-Neuberg Straße 1, Hannover, 30625 Germany; 2 School of Sports, Psychology and Education, Triagon Academy Munich, Ismaning, Germany; 3 Department of Health and Social Affairs, FHM Bielefeld - University of Applied Sciences, Bielefeld, Germany; 4 Institute of Biomechanics and Neurosciences, Nordic Science, Hannover, Germany

**Keywords:** Personality traits, Big five, Recreational handball

## Abstract

**Background:**

This study examines associations and group differences between personality traits, age, sex, performance level, and playing position in recreational handball players using the five-factor model.

**Methods:**

One hundred eighty-three handball players (114 female, 69 male; mean age = 26.4 ± 11.2 years), competing in leagues from 8 to 4th league, completed the NEO-FFI-30 in an online survey. Differences between sex, age groups, playing positions, and leagues were analyzed using t-tests and ANOVAs, with correlation and regression analyses examining relationships between traits and factors like age, handball experience, and weekly training.

**Results:**

Male players show lower neuroticism than female players (*p* < .001, *d* = 1.06). Senior players exhibited lower neuroticism (*p* < .001, *d* = 0.74) and higher conscientiousness (*p* = .006, *d* = -0.54) compared to youth players. Age predicted neuroticism (β = -0.15, *p* < .001), openness (β = 0.06, *p* = .022), and conscientiousness (β = 0.08, *p* < .001). Trainers showed significantly lower neuroticism (*p* = .023, *d* = -0.65) and higher openness compared to field players (*p* = .014, *d* = 0.69) and goalkeepers (*p* = .019, *d* = 0.9). Fourth-league players exhibited higher neuroticism than fifth-league (*p* = .036, *d* = 0.81) and sixth-league players (*p* = .042, *d* = 0.7). A small but significant positive correlation was found between extraversion and performance level (*r* = .152, *p* = .041). Conscientiousness predicted weekly handball training volume (β = -5.47, *p* = .012). Handball experience predicted neuroticism (β = -0.17, *p* < .001) and conscientiousness (β = 0.08, *p* < .001).

**Conclusions:**

Findings on age-related differences may help coaches better adapt their approach to players and distribute roles based on individual personality strengths and experience. However, sex-related comparisons should be interpreted cautiously due to differences in age and experience between male and female participants.

## Highlights

- Male players and senior athletes showed significantly lower neuroticism scores.
- Higher neuroticism was found in higher league players compared to lower leagues.
- Extraversion showed a positive correlation with performance level.
- Handball experience predicted lower neuroticism and higher conscientiousness.
- Coaches were more open and emotionally stable than players and goalkeepers.

## Introduction

Personality plays a fundamental role in shaping human performance, as different personality traits influence how individuals face challenges, set goals, and achieve success [18]. Research shows that these traits not only influence a person ‘s interactions and psychological adjustment but also their ability to master specific and demanding tasks [14]. The Big Five model, which is regarded as the global consensus for personality research, plays a particularly important role in this context [12, 34]. The Big Five framework has also been applied in handball contexts. Mihaela et al. [24] examined the five personality dimensions in junior handball players and reported associations between Big Five traits such as emotional stability and autonomy and cognitive characteristics related to sport behavior.

Personality as a psychological factor that influences performance has been presented in a variety of studies across sports [26, 29, 41]. Similar relationships between personality traits and social roles have also been observed in handball. Predoin et al. [33] found that traits such as extraversion, emotional stability, and conscientiousness were positively associated with the social status and informal leadership roles of players within handball teams. The results of this research show that low neuroticism, high extraversion, and high agreeableness can contribute to higher performance [13, 31, 42]. Low neuroticism, i.e. emotional stability, is particularly important, as it reduces anxiety and negative emotions [15]. Good performance is therefore all the more likely the lower the level of neuroticism [31]. In addition, increased activation can be observed in athletes with high neuroticism, which makes them more susceptible to stress and therefore more likely to be anxious before a competition [3]. Furthermore, a high level of conscientiousness is also discussed as being conducive to performance [3, 42]. In addition, the four personality dimensions mentioned also have a connection to competition anxiety, which is known to have a negative impact on an athlete ‘s performance [17].

When looking at different sports with different requirement profiles, Piepiora et al. [31] describe that extraversion and neuroticism play a major role in competitive athletes of all sports, while the other characteristics vary depending on the sport. Accordingly, there is a need for larger studies that look at the influence of personality traits on performance in a particular sport. This is particularly important because personality profiles are relevant for athletic performance and should therefore be considered in the sport psychological preparation of athletes [25].

At present, there is no known work that examines the personality traits of handball players in recreational sports and at the same time includes age, sex, performance level, playing and desired position, as well as training effort and playing experience. Previous research in handball on personality profiles has either been limited to individual groups such as professional referees [8], amateur referees [9] or goalkeepers [11] or only refers to individual sub-aspects, such as sex differences [28] or differences between age groups [31, 39]. The studies that investigated not only partial aspects but the entire correlations with personality traits, have so far only dealt with the professional sector and not with amateur players and were limited either only to men [36] or only to youth players [19, 20]. Further findings in the leisure sector can be used for a broad range of athletes and coaches for talent forecasting, prevention programs, individualization, and improving performance.

The aim of this study was to investigate the relationship between personality traits and (a) performance level, (b) sex, (c) age, and (d) playing or desired position in recreational handball. Based on existing research across various sports, it can be assumed that, as in other sports, low neuroticism is associated with better performance in handball. Accordingly, higher league levels are expected to be associated with lower levels of neuroticism. It is also hypothesized that goalkeepers tend to be more agreeable and more neurotic compared to other playing positions, consistent with findings by Fasold et al. [11] and Kőnig-Görögh et al. [19]. In addition, coaches were included in the sample for an exploratory secondary analysis aimed at comparing personality profiles between coaches, field players and goalkeepers, which to date has not been investigated in recreational handball.

## Methods

### Participants

Participants in the study were handball players (*n* = 217) and coaches (*n* = 19) from Germany, all of whom were at least 16 years old. In Germany, a total of 793,149 members are registered in the German Handball Federation (DHB), making the DHB the seventh-largest elite sports association in Germany, with the majority being active in the recreational sector [7]. Two handball players were excluded as they are not currently active in handball themselves. To minimize possible influencing factors on the personality profile, participants who reported an acute or past mental illness or the use of psychotropic drugs were also excluded from the study (*n* = 21). Furthermore, participants from the first three national leagues (i.e., professional handball payers) were not included in the sample (*n* = 11), as the study was intended to focus on the recreational sports sector. Thus, after applying the exclusion criteria, the data of 183 handball players (25 pivot players, 40 left/right wingers, 39 center backs, 57 left/right backs, and 22 goalkeepers) and 19 coaches were analyzed. Coaches were included for an exploratory comparison of personality profiles between coaches, field players, and goalkeepers. Participants ranged in age from 16 to 73 years and included both handball players and coaches. Table 1 presents the demographic and training characteristics of the handball players, including their handball experience, weekly training volume, and performance level. All participants took part in the survey voluntarily, were informed about the purpose of the survey for research purposes and agreed with publication. The data was collected anonymously.

**Table 1:** Descriptive statistics on age, handball experience, handball training volume per week, additional training per week, and performance level for players, broken down by sex

	**Total (mean ± SD)**	**Male (mean ± SD)**	**Female (mean ± SD)**
N	*183*	*69*	*114*
Age (years)	26.44 ± 11.23	31.06 ± 13.68	23.51 ± 8.18
Handball experience in years	17.16 ± 10.26	20.54 ± 11.74	14.83 ± 7.97
Handball training volume in min/week	234.12 ± 90.45	214.28 ± 68.10	246.93 ± 103.52
Athletic training volume in min/week	98.60 ± 90.19	91.30 ± 87.51	103.25 ± 92.21
Performance level	2.53 ± 1.46	2.17 ± 1.24	2.77 ± 1.59

Performance level: 1 = 8th league(lowest amateur level) to 5 = 4th league (highest amateur level)

To estimate the required sample size for detecting meaningful effects within our study, a power analysis was conducted based on previously reported effect sizes in the literature. Kőnig-Görögh et al. [19] reported various effect sizes using Cohen's *d*, ranging from 0.88 for conscientiousness differences between medalists and non-medalists to 1.96 for openness. These substantial effect sizes suggest significant variability in personality traits that could potentially be linked to performance and other demographic factors. For our power analysis, we chose to use a conservatively estimated medium effect size (Cohen's *d* = 0.5), as reported in numerous personality studies within sports psychology [4]. This decision was informed by our desire to robustly detect smaller but still meaningful differences between groups (e.g., by sex, age, and playing position) with sufficient statistical power. To achieve 80% power at a significance level (*α*) of .05, the calculations indicated that approximately 64 participants were needed.

### Procedures

To address the research questions of this study, handball players and coaches from leagues ranging from the German Kreisliga to Oberliga, aged 16 years and older, were surveyed using an online questionnaire assessing personality traits according to the five-factor model. The data was collected between 05.08.2024 and 26.10.2024 using an online questionnaire, which was created and published using the empirio.de platform. A total of 68 handball clubs from the included amateur leagues were contacted and asked to distribute the survey link among their players. Additionally, handball coaches and regional federation representatives known to the authors were asked to forward the link, and the study was also promoted via social media. Two weeks before the end of the data collection period, a reminder to participate was sent to all relevant representatives. The questionnaire was designed in such a way that every single question had to be answered so that only complete surveys were sent for evaluation. Furthermore, multiple participants from the same end device were excluded from the survey by an IP blocking algorithm provided by the online platform. In addition to demographic data (age and sex), the questionnaire asked about performance level, training volume per week (in minutes), divided into handball training and athletic training, and handball experience in years. Furthermore, the current playing positions and the player's preferred position were recorded. The playing positions available for selection were right/left back, left/right wing, center back, pivot, goalkeeper, and coach. In addition, current and past mental illnesses and the use of psychotropic drugs were also queried.

The German version of the NEO-FFI-30 was used to assess personality traits according to the five-factor model. The 30-item questionnaire measures neuroticism, extraversion, openness, agreeableness, and conscientiousness using a five-point Likert scale and shows high agreement with the original NEO-FFI (ICC = .91–.95) [6] as well as satisfactory internal consistency (Cronbach’s α = .67–.81) [21].

### Statistical analysis

The statistical analysis was carried out using Jamovi (www.jamovi.org, version 2.3.26). Descriptive statistics were used to illustrate the participant characteristics. Differences in personality traits between sex, age groups (youth vs. senior), playing positions, and performance levels were analyzed using t-tests and ANOVAs, supplemented by Tukey post-hoc tests for significant ANOVA results. Correlations between personality dimensions and performance level or training volume (in minutes) were recorded using Spearman and Pearson correlations. Simple linear regression analyses were conducted to examine whether age and years of handball experience predicted personality traits. In addition, a multiple linear regression model was used to test whether the five personality traits predicted weekly handball training volume. The normality of the data distribution was assessed using the Shapiro–Wilk test. Although minor deviations from normality were observed, the analyses were considered robust due to the sample size [45]. The variance homogeneity was given for almost all calculations (Levene's test without significant results, *p* > .05). Only in the t-test for the comparison of sex on the neuroticism characteristic was the variance homogeneity not fulfilled, which is why in this case the less susceptible Welch's t-test was calculated instead of the Student's t-test. Cohen's d was calculated for all t-tests and single-factor analyses of variance to indicate the effect size. According to Cohen [4], *d* = 0.2 represents a small effect, *d* = 0.5 a moderate effect and *d* = 0.8 a large effect. For all correlations, the calculated correlation coefficient *r* was interpreted according to the scheme by Vincent and Weir [44]. This designates 0 ≤ *r* ≤ .69 as a low correlation, .70 ≤ *r* ≤ .89 as a moderate correlation, and *r* ≥ .90 as a strong correlation. A *p*-value of < .05 was considered significant for all test procedures.

## Results

### Age and sex differences

**Fig. 1:**
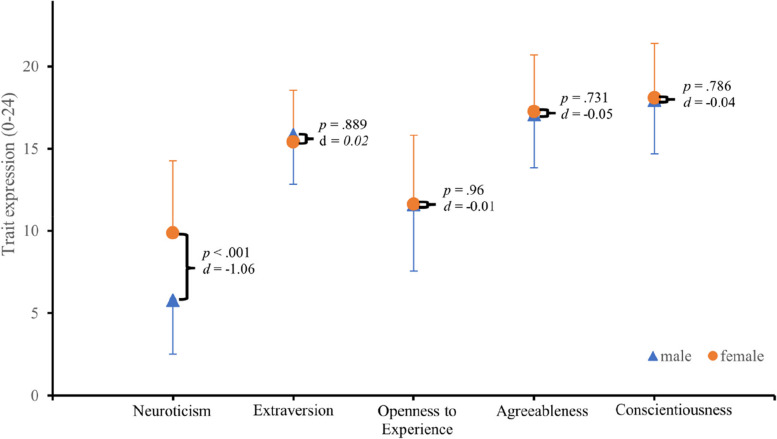
The five personality traits in comparison between male and female handball players

The t-tests carried out showed that there were no significant differences between sexes for the characteristics extraversion (*p* = .889, *d* = 0.02), openness to new experiences (*p* = .96, *d* = −0.01), agreeableness (*p* = .731, *d* = −0.05) and conscientiousness (*p* = .786, *d* = −0.04) (Fig. 1). Only for the personality trait neuroticism was it found that male players on average showed significantly lower neuroticism (*M* = 5.77, *SD* = 3.25) than female players (*M* = 9.86, *SD* = 4.41), *t*(174) = 7.19, *p* < .001, *d* = 1.06, indicating a large effect (Fig. 1).

**Fig. 2:**
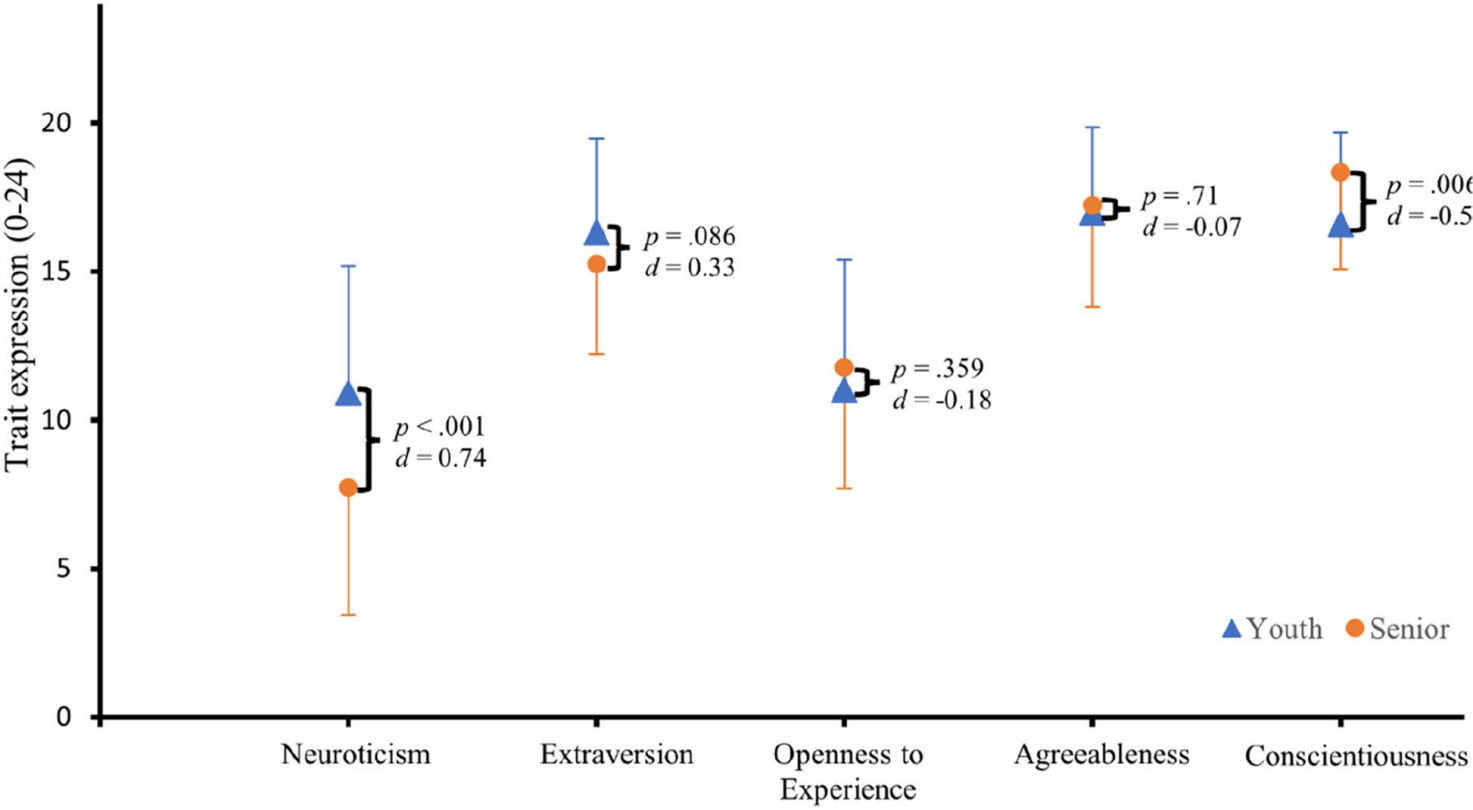
The five personality traits in comparison between youth and senior players

Figure 2 shows the average values of the personality traits for youth and senior players in comparison. On average, the senior players showed significantly lower neuroticism (*M* = 7.74, *SD* = 4.31) than the youth players (*M* = 10.9, *SD* = 4.29), *t*(181) = 3.86, *p* < .001, *d* = 0.74, and higher conscientiousness (*M* = 18.34, *SD* = 3.27) than the youth players (*M* = 16.6, *SD* = 3. 07), *t*(181) = 2.79, *p* = .006, *d* = −0.54, both indicating a moderate effect. No significant differences were found between youth and senior players for the characteristics of extraversion (*p* = .086, *d* = 0.33), openness to new experiences (*p* = .359, *d* = 0.18), and agreeableness (*p* = .71, *d* = 0.07).

The regression model predicting neuroticism from age can be expressed as: neuroticism = *β*₀ + *β*₁(age). In addition, the regression analyses revealed a significant overall model for the prediction of neuroticism, *F*(1, 181) = 30.2, *p* < .001, openness to new experiences, *F*(1, 181) = 5.32, *p* = .022, and conscientiousness, F(1, 181) = 15.9, *p* < .001 by age. The distribution of residuals did not deviate significantly from normality (Shapiro–Wilk test, *p* > .05). Overall, the models explained 14.3% (for neuroticism), 2.86% (for openness), and 8.08% (for conscientiousness) of the variance in the traits. It was also shown that age contributes significantly to the variance explanation of neuroticism (*β* = −0.15, *p* < .001), openness to new experiences (*β* = 0.06, *p* = .022), and conscientiousness (*β* = 0.08, *p* < .001). No significant overall models were found for the prediction of Extraversion (*p* = .586) and Agreeableness (*p* = .19) by age.

### Differences between the playing and desired positions

The analysis of variance showed no significant effects of playing or desired positions on any of the five personality dimensions (Table 2). No significant differences were found between positions, including left/right back, center back, wing, pivot, and goalkeeper.

**Table 2:** Results of the single-factor ANOVA for the differences in personality traits in the playing positions and in the desired positions

*Trait*	**Playing Position (** ***F*** **)**	**Playing Position (** ***p*** **)**	**Desired Position (** ***F*** **)**	**Desired Position (** ***p*** **)**
*Neuroticism*	1.719	.133	0.079	.989
*Extraversion*	0.517	.763	0.912	.458
*Openness*	1.867	.102	1.147	.336
*Agreeableness*	0.475	.795	1.370	.246
*Conscientiousness*	1.939	.090	1.008	.405

^*^ = significant (*p* <.05)

### Differences between players, goalkeepers and coaches

**Fig. 3:**
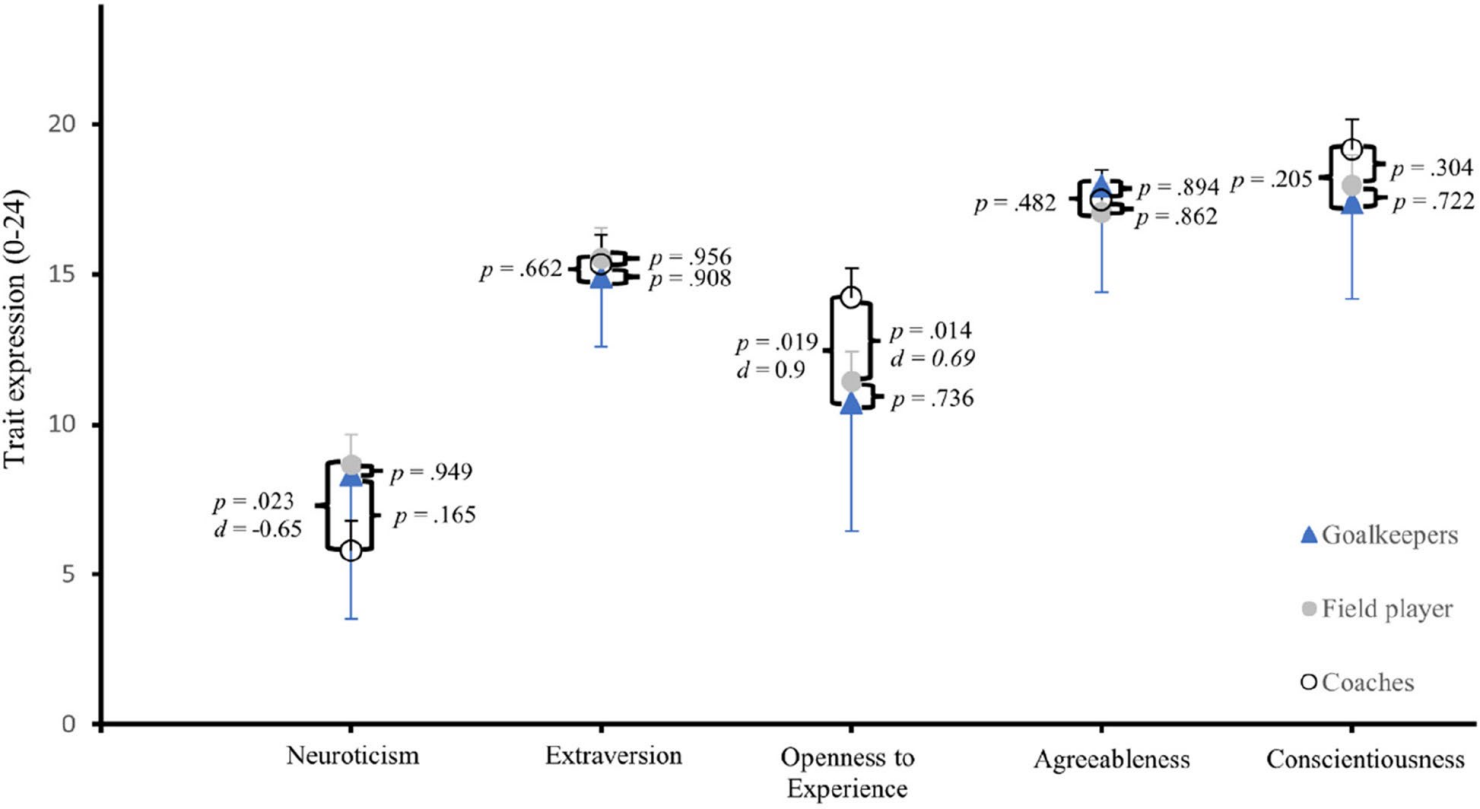
The five personality traits compared between goalkeepers, field players, and coaches. *Note.* The effect size *d* was only given for significant results

When comparing the three groups of coaches, field players, and goalkeepers, significant main effects were found for neuroticism, *F*(2, 180) = 3.53, *p* = .031, and openness, *F*(2, 180) = 4.6, *p* = .011 (Fig. 3). Tukey post-hoc tests revealed that coaches scored significantly lower in neuroticism (*M* = 5.79, *SD* = 3.33) than field players (*M* = 8.65, *SD* = 4.47) (*p* = .023, *d* = −0.65), indicating a moderate effect, with no significant differences between coaches and goalkeepers (*p* = .165) or field players and goalkeepers (*p* = .949). Coaches were also significantly more open to new experiences (*M* = 14.21, *SD* = 3.21) than both field players (*M* = 11.42, *SD* = 4.1) (*p* = .014, *d* = 0.69) and goalkeepers (*M* = 10.71, *SD* = 4.28) (*p* = .019, *d* = 0.9), while no significant difference was found between field players and goalkeepers in openness (*p* = .736).

### Differences and correlations in performance levels

The one-way ANOVA for the comparison of personality traits between the performance levels 8th league (lowest amateur level) to 4th league (highest amateur level) revealed significant main effects for the traits neuroticism, *F*(4, 178) = 2.84, *p* = .026 and agreeableness, *F*(4, 178) = 2.62, *p* = .037. The subsequent Tukey posthoc tests revealed that, on average, 4th league handball players exhibited significantly higher neuroticism (*M* = 10. 92, *SD* = 4.71) compared to 5th league (*M* = 7.69, *SD* = 4.4) (*p* = .042, *d* = 0.7) and also 6th league handball players (*M* = 7.36, *SD* = 3.89) (*p* = .036,* d* = 0.81). The comparisons of all other performance levels revealed no significant differences in terms of neuroticism in the leagues (Table 3). The post-hoc tests for agreeableness revealed no significant differences between the individual performance levels despite the significant overall model (Table 4).

**Table 3:** Post-hoc tests for the neuroticism trait in comparison between the performance levels

Performance level *MD* (*p*)	8th league	7th league	6th league	5th league	4th league
8th league	-	0.62 (.97)	0.94 (.89)	0.61 (.96)	−2.62 (.08)
7th league	-	-	0.32 (1.00)	0.01 (1.00)	−3.24 (.06)
6th league	-	-	-	−0.33 (1.00)	−3.56 (.04)*
5th league	-	-	-	-	−3.23 (.04)*
4th league	-	-	-	-	-

Performance level: 8th league = lowest amateur level, 4th league = highest amateur level

^*^ = significant (*p* <.05)

**Table 4:** Post-hoc tests for the agreeableness in comparison between the performance levels

Performance level MD (p)	8th league	7th league	6th league	5th league	4th league
8th league	-	1.84 (.09)	−0.38 (.99)	0.36 (.99)	1.50 (.29)
7th league	-	-	−2.22 (.11)	−1.49 (.39)	−0.34 (1.00)
6th league	-	-	-	0.74 (.91)	1.88 (.26)
5th league	-	-	-	-	1.14 (.67)
4th league	-	-	-	-	-

Performance level: 8th league = lowest amateur level, 4th league = highest amateur level

^*^ = significant (*p* <.05)

The correlation analysis led to a low positive correlation between the trait extraversion and the performance level, *r*(181) = .152, *p* = .041. No significant correlations were found for the traits neuroticism (*p* = .228), openness to new experiences (*p* = .085), agreeableness (*p* = .308), and conscientiousness (*p* = .378) with the performance level.

### Correlations between training volume and handball experience

The correlation analysis showed no significant relationships between additional training volume and the five personality traits (neuroticism: *p* = .795, extraversion: *p* = .494, openness: *p* = .96, agreeableness: *p* = .397, conscientiousness: *p* = .5). Similarly, no significant correlations were found between handball training volume and neuroticism (*p* = .235), extraversion (*p* = .151), openness (*p* = .169), and agreeableness (*p* = .479). However, a small negative correlation was observed between conscientiousness and training volume (*r*(181) = −.199, *p* = .007).

The regression model predicting weekly handball training volume can be expressed as: training volume = *β*₀ + *β*₁(neuroticism) + *β*₂(extraversion) + *β*₃(openness) + *β*₄(agreeableness) + *β*₅(conscientiousness). Regression analysis yielded a significant model predicting handball training volume from personality traits, *F*(5,177) = 2.6, *p* = .027, explaining 6.85% of the variance. Conscientiousness contributed significantly (β = −5.47, *p* = .012), while other traits did not (neuroticism: *p* = .357, extraversion: *p* = .066, openness: *p* = .255, agreeableness: *p* = .945).

The regression model predicting neuroticism from handball experience can be expressed as: neuroticism = *β*₀ + *β*₁(handball experience). Regression analyses also showed that years of handball experience predicted neuroticism, *F*(1, 181) = 30.0, *p* < .001, explaining 14.2% of its variance, and conscientiousness, *F*(1, 181) = 12.1, *p* < .001, explaining 6.25%. Experience was a negative predictor of neuroticism (β = −0.17, *p* < .001) and a positive predictor of conscientiousness (β = 0.08, *p* < .001). Extraversion (*p* = .425), openness (*p* = .11), and agreeableness (*p* = .854) were not significantly predicted by handball experience.

## Discussion

The present study examined the relationship between Big Five personality traits and demographic as well as sport-related factors in recreational handball players. The following discussion interprets these findings while considering the exploratory nature of the study and the demographic differences between the analyzed subgroups.

### Age and sex differences

Youth and senior players differed significantly in neuroticism (*d* = 0.74) and conscientiousness (*d* = −0.54), with seniors displaying lower neuroticism and higher conscientiousness. This finding may align with Tedesqui and Young [43], who link higher conscientiousness to increased experience and skill in athletes. While Piepiora et al. [31] found no personality trait differences between youth and adult elite athletes, suggesting stabilization at high competition levels, the current study’s focus on recreational players may reveal more pronounced age-related differences. Regression analyses also confirmed that neuroticism decreases and conscientiousness increases with age. When comparing the observed personality scores with population-based reference values for the NEO-FFI, the overall pattern in the present sample appears broadly consistent with normative ranges reported for adult populations [21]. However, the comparatively lower neuroticism observed in older players may reflect psychological adaptations associated with long-term sport participation [30]. Additionally, openness increased with age, potentially reflecting greater self-confidence and receptivity to new approaches among older players.

Contrary to Kőnig-Görögh et al. [19, 20], who reported higher neuroticism in males and greater conscientiousness and openness in females, our study found that males displayed significantly lower neuroticism (*d* = 1.06) than females. This discrepancy may result from differences in age and competitive levels between studies. Paušek et al. [28] also observed higher extraversion in males but noted reliability issues with their assessment tool. However, these sex-related findings should be interpreted with caution, as the male and female subgroups in the present study differed noticeably in age and handball experience.

### Differences between playing and desired positions

No significant differences were found in personality dimensions across either playing or desired positions for field players and goalkeepers, which is surprising, particularly for desired roles, as personality profiles might be expected to align with position preferences. Given the limited research on personality and preferred positions, further studies would help clarify these relationships. Additional research could also explore how alignment or mismatch between actual and desired positions influences player satisfaction and potentially performance, especially at higher levels.

This study aligns with Kőnig-Görögh et al. [20], refuting the hypothesis that goalkeepers have higher agreeableness and neuroticism as suggested by Kőnig-Görögh et al. [19] and Fasold et al. [11]. Fasold et al. [11] focused solely on male goalkeepers in comparison with the general population, which may explain the different outcomes. Kőnig-Görögh et al. [19] also examined younger, elite athletes, potentially accounting for their marginally significant differences. Additionally, Rogulji et al. [36] found higher extraversion among professional outfield players than goalkeepers and backcourt players, suggesting that observed personality differences in playing positions might primarily reflect the professional sector rather than recreational levels.

### Differences between players, goalkeepers and coaches

Meaningful differences were observed between the three groups of field players, goalkeepers, and coaches. Coaches showed lower neuroticism than field players (*d* = −0.65), consistent with the need for emotional stability to lead teams effectively in challenging situations. Coaches also scored significantly higher in openness compared to field players (*d* = 0.69) and goalkeepers (*d* = 0.9), reflecting the adaptability and willingness to embrace new approaches necessary for planning, training, and tactical guidance [27]. This openness is fitting for the demands of coaching, where continual self-reflection and adaptation to new knowledge are crucial [5].

### Differences between performance levels

In examining performance levels, 4th league handball players demonstrated higher neuroticism on average than 5th and 6th league players, with a moderate effect (*d* = 0.7) between the 4th and 5th leagues and a large effect (*d* = 0.81) between the 4th and 6th leagues. This was unexpected, as higher emotional stability was anticipated at higher competitive levels. Previous research has indicated that lower neuroticism is generally beneficial for sports performance, as it correlates with greater emotional stability and resilience under pressure [13, 30, 42]. These findings are also consistent with broader evidence showing that personality traits such as conscientiousness and extraversion are positively associated with athletic performance and competitive success across various sports disciplines [1, 22]. One possible explanation for the higher neuroticism observed among higher-league players could be that competitive demands and performance expectations may differ across amateur league levels, even within recreational sport contexts. This recreational orientation may be associated with lower performance-related pressure and, consequently, lower neuroticism [46]. In light of these findings, Li et al. [23] suggested that incorporating psychological skills training even among recreational athletes may help mitigate elevated neuroticism and enhance emotional resilience.

A small positive correlation between extraversion and league level was also observed, which aligns with findings that extraversion is advantageous in team sports where effective communication, quick decision-making, and dynamic interpersonal engagement are critical [16, 38]​. Extraverted athletes tend to foster an energetic team atmosphere, enhancing collective performance, a trait particularly valuable in interactive sports like handball [37].

Although conscientiousness did not emerge as a significant factor in this recreational sample, it has been identified as a predictor of athletic success in professional team sports, attributed to qualities like self-discipline, responsibility, and reliability [2, 16]. This distinction may highlight differences in trait relevance between recreational and professional athletes, as conscientiousness plays a more pronounced role in high-performance, competitive contexts [42].

### Differences between training volume and handball experience

The analysis revealed a slight negative correlation between conscientiousness and handball training volume, with regression analysis confirming that lower conscientiousness predicts higher training volume. Although this finding seems counterintuitive, since conscientiousness often relates to diligence and consistency [10], training volume in team sports typically depends on scheduled team sessions rather than individual choice. Thus, players' participation may hinge on factors beyond personal conscientiousness. Further studies with larger samples are needed to examine if this relationship holds across different contexts.

Additionally, regression analysis showed that handball experience significantly predicts neuroticism and conscientiousness levels, with increasing experience, neuroticism decreases while conscientiousness rises. This aligns with expectations, as experience enhances emotional stability [30] and fosters the discipline and reliability needed for team responsibility [42]. These findings support the notion that experience in team sports can positively shape personality traits over time. At the same time, the relationship between personality traits and sport participation is likely bidirectional. While long-term participation in structured training environments may contribute to gradual personality development, personality traits may also influence individuals’ engagement and persistence in sport [2]. In addition, a selection effect cannot be ruled out, as individuals with traits such as higher conscientiousness or emotional stability may be more likely to remain active in handball over longer periods. Similar patterns have been reported in athlete–non-athlete comparisons, where athletes often display higher extraversion and conscientiousness and lower neuroticism than non-athletes [35].

### Limitations

This study has several limitations. Firstly, the sample included significantly more female than male handball players (114 F, 69 M), reflecting response rates rather than the actual sex distribution in recreational handball, which may have influenced some parameter evaluations. In addition, differences in age and weekly training volume between male and female participants may have influenced group comparisons. Furthermore, the relatively large standard deviations observed in several variables indicate a certain heterogeneity within the sample, which should be considered when interpreting the results. This imbalance may be explained by previously reported sex differences in participation behavior in psychological surveys, with female athletes generally showing greater willingness to participate in online studies related to personality and mental attributes [32, 40]. Additionally, the recruitment process through club networks may have unintentionally favored female-dominated teams.

Furthermore, district league players were overrepresented compared to other leagues. However, this can still be considered representative, as the district league, being the lowest division, likely includes a larger active player base. Similarly, comparisons between coaches, outfield players, and goalkeepers showed uneven group sizes due to the naturally higher number of outfield players. All analyses were conducted on the full sample, partly due to sample size constraints and the group distributions mentioned above.

## Conclusion

This study examined the relationship between Big Five personality traits and demographic as well as sport-related factors in recreational handball players, including sex, age, performance level, playing position, and handball experience. The findings revealed notable differences related to sex and age. Senior players displayed lower neuroticism and higher conscientiousness than youth players, suggesting that age and handball experience may contribute to greater emotional stability and a more disciplined approach to training. Additionally, distinct personality traits were observed among coaches, field players, and goalkeepers. Coaches demonstrated lower neuroticism and higher openness, traits that likely support their roles in leadership and adaptability under pressure, while field players and goalkeepers showed consistent profiles within their respective positions.

These findings underline the potential for using personality assessments in recreational handball to enhance team dynamics, optimize player roles, and better align training approaches with individual traits. By understanding the personalities within a team, coaches may tailor their communication, assign responsibilities that align with personal strengths, and create a supportive environment that encourages both individual growth and team cohesion.

For future research, larger and more diverse samples across different league levels and positions are recommended to deepen understanding of personality's impact in recreational sports. This could further clarify the relationships between personality, performance, and training commitment, offering practical applications for player development, team strategies, and recreational sports psychology.

## Data Availability

The original contributions presented in the study are included in the article/supplementary material, further inquiries can be directed to the corresponding author/s.
